# Suicidal Behaviors Among Medical Students: A Scoping Review of Systematic Reviews and Meta-Analyses

**DOI:** 10.3390/bs15091215

**Published:** 2025-09-07

**Authors:** Felix Agyapong-Opoku, Nadine Agyapong-Opoku, Belinda Agyapong, Andrew Greenshaw

**Affiliations:** 1 School of Medicine, University of Galway, H91 TK33 Galway, Ireland; 2 Department of Psychiatry, University of Alberta, Edmonton, AB T6G 2H5, Canada; nagyapon@ualberta.ca (N.A.-O.); bagyapon@ualberta.ca (B.A.); andrew.greenshaw@ualberta.ca (A.G.); 3 College of Health Sciences, University of Ghana Medical School, Accra P.O. Box GP 4236, Ghana

**Keywords:** suicidal ideation, suicide attempts, medical students, mental health, prevalence, risk factors, depression, burnout, academic stress

## Abstract

Background: Suicidal ideation and attempts are major public health concerns among young adults, particularly those in demanding academic settings. Medical students exhibit disproportionately high rates compared to peers in the general population and other fields of study, highlighting the urgent need to understand and address mental health challenges in medical education. Objective: This scoping review summarizes evidence from systematic reviews and meta-analyses on the prevalence and risk factors of suicidal ideation and suicide attempts among medical students worldwide. Methods: Following PRISMA-ScR guidelines, six databases were searched for peer-reviewed reviews published in the last ten years. Studies focused exclusively on medical students and reporting prevalence or risk factors of suicidal ideation or attempts were included. Data were charted on prevalence, risk factors, study characteristics, and recommendations. Results: Twelve reviews comprising 378,081 medical students were included. Lifetime prevalence of suicidal ideation ranged from 2.9% to 53.6% among the systematic reviews, with pooled estimates from meta-analyses ranging from 11% and 25%. Attempted suicide pooled prevalences ranged from 1.64% to 8%. Depression was frequently reported as the most significant risk factor for both suicidal ideation and attempts. Other significant risk factors for suicidal ideation included anxiety, burnout, female gender, financial strain, and academic stress. Suicidal ideation was higher during the COVID-19 pandemic and among clinical-phase students. Gender differences in suicide attempts were inconsistent. Medical students’ rates of suicidal behavior exceeded those of other university students. Conclusion: Suicidal behavior remains a critical mental health issue for medical students globally. Despite known risk factors, targeted interventions are limited. Future research should emphasize longitudinal studies, post-pandemic effects, regional gaps, and intervention development. Implications are discussed.

## 1. Introduction

Suicidal behavior can encompass a broad range of behaviors including suicidal thoughts (ideations), suicidal planning, and suicidal attempts (World Health Organization, 2014). Suicide is the deliberate, often premeditated, tragic action of ending one’s own life, and it is often carried out amid severe psychological distress when individuals feel a sense of hopelessness (Harmer et al., 2025). Suicidal ideation refers to thoughts of self-harm or ending one’s life, often associated with mental health disorders or situational stressors, and requires timely recognition and intervention to prevent the drastic action of suicide (Harmer et al., 2025). Suicidal ideation is a major risk factor associated with future suicide attempts (Han et al., 2016). Suicidal ideations can stem from a combination of psychological, social, and situational factors, highlighted by research over the past decade which includes the significant role of psychological strain and mediating factors (Sun et al., 2020; Zhang et al., 2018), individual’s attachment style (Sharif & Akhtar, 2018), burnout (Ishak et al., 2013; Kabir et al., 2023; Menon et al., 2020; Oh et al., 2023; Ryan et al., 2023), demoralization (Xu et al., 2019), bereavement (Molina et al., 2019), depression (Höller & Forkmann, 2022; Kim et al., 2021), alcohol consumption (García-Ramírez et al., 2025; Kim et al., 2021), diverse stressors, and dissatisfaction of the present, past, or future (Kim et al., 2021), among others. Suicidal behavior, though not classified as a mental health disorder, is frequently linked to mental health disorders as well as situational stressors (Harmer et al., 2025).

Medical students represent a uniquely vulnerable population when it comes to mental health concerns such as suicidal ideation (Dyrbye et al., 2006). Medical school is widely recognized as both academically and emotionally demanding, often characterized by high levels of stress, intense academic pressure, emotional fatigue, along with exposure to human suffering (Agyapong-Opoku et al., 2023; Dyrbye et al., 2006). These stressors may contribute to a level of psychological distress that can manifest as depression or suicidal ideation (Dyrbye et al., 2005; Mateen et al., 2024). Despite their medical knowledge and, in many settings, relatively greater access to mental health resources compared to the general population, medical students often do not seek professional help due to stigma, fear of professional repercussions, or a perceived need to appear resilient (Dyrbye et al., 2005). This suggests that the prevalence of mental health crises may be both under-reported and under-managed, which can further exacerbate the symptoms and worsen outcomes.

Globally, suicide is a leading cause of death among young adults aged 15–29 (Harmer et al., 2025; World Health Organization, 2016, 2025), and its prevalence among medical students appears to be disproportionately high compared to age-matched peers in the general population and students in other academic disciplines (Ikhsan et al., 2022; Mateen et al., 2024; Van Niekerk et al., 2012). Estimates of the prevalence of suicidal ideation among this population vary greatly across different studies, potentially resulting from differences in sample characteristics and cultural contexts, with systematic reviews portraying differences in ranges across their included studies (R. Coentre & Góis, 2018; Meireles Campos & Aleluia, 2019). The difference in prevalence according to different variables is important to investigate to better inform risk assessments.

A wide range of risk factors of suicidal ideation and attempts in this demographic have been researched, encompassing various psychological, interpersonal, and sociodemographic domains, among others (Seo et al., 2021). Examples include experiencing academic difficulty, depression, and being from a low-income status family. Published literature reflects the complexity of the issue, emphasizing that suicidal behavior among medical students cannot be attributed to a single cause.

Given the high stakes involved in both medical students’ well-being and future patient care, assessing the existing evidence on suicidal behavior within this population is crucial. A comprehensive understanding of the prevalence and associated factors can help educational policymakers recognize the scope of the issue and respond appropriately. It also allows for the identification of at-risk groups and the underlying drivers of distress.

Thus, the current scoping review will summarize existing evidence from systematic reviews and meta-analyses on the prevalence and risk factors of suicidal ideation and attempts among medical students globally. By mapping the current literature, this review seeks to clarify the extent of the problem, identify common risk factors, and highlight gaps in knowledge. The findings may help guide future research and inform the development of targeted interventions to address suicidal behavior within medical education.

## 2. Methodology

### 2.1. Study Design

This review was planned and conducted in adherence to the Preferred Reporting Items for Systematic Reviews and Meta-Analyses guidelines for scoping reviews (Page et al., 2021; Tricco et al., 2018). This review was guided by Arksey and O’Malley’s five-stage guide to conducting scoping reviews (Arksey & O’Malley, 2005) as follows: (1) developing a research question, (2) identifying relevant studies, (3) selecting articles, (4) charting of the data, and (5) collating and reporting of results. There is no published protocol for this review. The details of the systematic search are available in this manuscript.

### 2.2. Identifying the Research Questions

The aim of this scoping review is to examine evidence from systematic reviews and meta-analyses on the prevalence and associated factors of suicidal ideation and attempts among medical students globally in order to determine the scale of the problem and which variables are associated with increased risk of non-fatal suicidal behavior for students.

### 2.3. Identifying Relevant Studies

Five databases were searched to acquire relevant articles, using the same set of search terms for each: MEDLINE (Medical Literature Analysis and Retrieval System Online; Ovid MEDLINE ALL), CINAHL (Cumulative Index of Nursing and Allied Health Literature), APA PsycINFO (Ovid interface), PubMed (Public Medline), EMBASE (Excerpta Medica Database; Ovid interface), and Scopus Elsevier. The database search was concluded on the 15 May 2025.

The search used specific terms that were synonyms of the primary concepts such as prevalence, risk factors, medical students, and suicidal behaviors. The exact search terms were as follows: (Prevalence OR incidence OR epidemiology OR frequency OR occurrence OR rate) OR (Correlates OR associated factors OR predictors OR risk factors) AND (medical students OR students in medicine) AND (suicidal ideation OR suicidal behavior OR suicidal thoughts OR suicide attempt OR suicidality) AND (Systematic review OR Meta-analysis).

### 2.4. Selecting Articles

Inclusion criteria were used to develop a comprehensive search strategy. Eligible articles focused on prevalence and/or risk factors of suicidal ideation and/or suicide attempts among medical students. Only peer-reviewed systematic reviews or meta-analyses that were in English were included. Articles of all other types were excluded. Interventional studies were excluded. In addition, articles were excluded if they did not have a sample of exclusively medical students. The search was limited to articles published within the last 10 years. This ensured that the review prioritized the most current and contextually relevant evidence, reflecting contemporary trends in medical education and medical student mental health.

**Figure 1:**
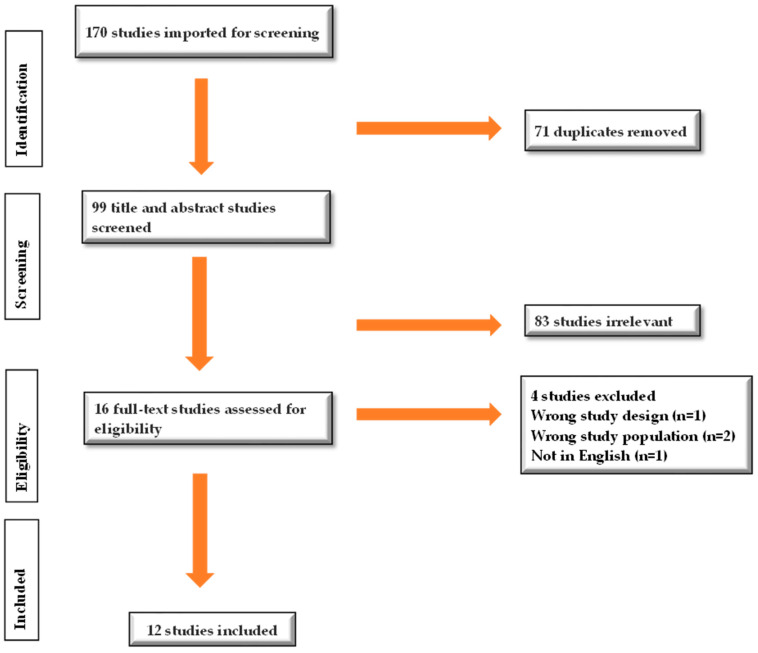
PRISMA flowchart for included and excluded studies.

Two independent researchers (F.A.O. and N.A.O.) reviewed and screened the compiled studies during both title and abstract and full text screening. The Covidence software was used as the screening tool for this scoping review. A total of 170 articles were identified from the databases using the search strategy. Seventy-one duplicates were automatically removed by the Covidence software. After title and abstract screening of the 99 articles, sixteen remained for full text screening, twelve of which were ultimately included. Conflicts were resolved during the title/abstract and full text screening after discussion and consensuses were reached. The PRISMA flow diagram in Figure 1 portrays the details of the screening process.

### 2.5. Charting Data

Data was extracted by the researchers for each article that met the inclusion criteria according to the following fields: author(s) name, year of publication, time period researched, number of included articles, continent of study, aim of review, participant characteristics and sample size (N), bias assessment, associated factors, key finding on prevalence, other significant finding, and recommendations. These fields were designed to ensure that each article was comprehensively summarized to address the research question. The summarized data from each study was validated by two researchers to ensure the information was accurate (F.A.O. and N.A.O.).

### 2.6. Collating, Summarizing, and Reporting Results

This scoping review gives a summary of existing data on the prevalence and risk factors of suicidal ideation and attempts among medical students from the past ten years. Pertinent data was organized into tables and confirmed to be valid by two researchers. Details and results of each included article are presented in Table 1. The results from the included articles in this review were narratively summarized, and quantitative synthesis or statistical summary measures were not used.

## 3. Results

A total of twelve studies were ultimately included. The total sample size across the included studies was 378,081. The sample sizes ranged from 1611 to 215,880 medical students. The number of relevant primary studies within the reviews ranged from 3 to 88. Seven of the reviews included studies from a range of countries and continents without a particular focus on any geographical region (R. Coentre & Góis, 2018; R. M. Coentre & Figueira, 2015; Mateen et al., 2024; Peng et al., 2023; Rotenstein et al., 2016; Seo et al., 2021; Tsegay et al., 2020). Some articles focused on a specific country—India (Kaur et al., 2024), Bangladesh (Rahman et al., 2025), China (Wang et al., 2023; Zeng et al., 2019)—or continent—Africa (Kaggwa et al., 2023). The continents of the primary studies were Europe, North America, Asia, Africa, and South America.

Table 1 gives detailed information about the included articles. In total, 3 of the 12 included articles were systematic reviews, two articles were meta-analyses, and seven articles were both systematic reviews and meta-analyses. Of the 12 reviews, two reviews (R. Coentre & Góis, 2018; R. M. Coentre & Figueira, 2015) provide a range for their primary finding of prevalence, while eight reviews (Kaggwa et al., 2023; Kaur et al., 2024; Peng et al., 2023; Rahman et al., 2025; Rotenstein et al., 2016; Tsegay et al., 2020; Wang et al., 2023; Zeng et al., 2019) offered a pooled prevalence summary based on meta-analyses. Additionally, two of the reviews (R. Coentre & Góis, 2018; Mateen et al., 2024) give only a narrative summary of risk factors, whilst three articles (Kaggwa et al., 2023; Rahman et al., 2025; Seo et al., 2021) conducted meta-analyses.

Only one article (Tsegay et al., 2020) of the 12 articles primarily focuses on suicide attempts; however, four articles (Kaggwa et al., 2023; Rahman et al., 2025; Seo et al., 2021; Wang et al., 2023) included information on suicide attempts in addition to suicidal ideation. Seven articles (R. Coentre & Góis, 2018; R. M. Coentre & Figueira, 2015; Kaur et al., 2024; Mateen et al., 2024; Peng et al., 2023; Rotenstein et al., 2016; Zeng et al., 2019) focused solely on suicidal ideation.

### 3.1. Prevalence of Suicidal Ideation Among Medical Students

The prevalences of suicidal ideation varied quite drastically across different medical school populations. For example, the lifetime frequency of suicidal thoughts among medical students worldwide varied from 2.9% to 53.6% among the included studies, according to R. Coentre and Góis (2018). Some of the reviews generally differed in the period of suicidal ideation explored (lifetime, past 12 months, etc.), which contributed to some of the differences in the prevalences investigated. All the articles that investigated suicidal ideation also showed a wide variation in prevalence among individual primary research articles. Among the meta-analyses, this resulted in considerably high heterogeneity scores.

The range of pooled prevalences across the meta-analyses was 11% to 25% (Rahman et al., 2025; Zeng et al., 2019). One meta-analysis focusing on African students reported a lifetime prevalence of 18.7% (Kaggwa et al., 2023). Among Bangladesh medical students, Rahman et al. (2025) found a lifetime prevalence of 25%. A prevalence of 21% was reported by Kaur et al. (2024) among Indian medical students. In a recent review involving 88 studies, Wang et al. (2023) reported a 13% prevalence of suicidal ideation among Chinese medical students. Zeng et al. (2019) previously observed a similar rate of 11%.

When studies from different countries and continents were combined across a global sample, R. M. Coentre and Figueira (2015) reported a prevalence range of 4.4% to 23.1% and R. Coentre and Góis (2018) gave a range of 2.9% to 53.6% in a later review. Rotenstein et al. (2016) showed in a meta-analysis that 11.1% of medical students had suicidal ideation over the past 12 months among a diverse range of countries. In a study of the prevalences during COVID-19 pandemic, Peng et al. (2023) showed a pooled prevalence of 15%.

### 3.2. Prevalence of Suicidal Attempt Among Medical Students

Only one of the articles strictly focused on prevalence of suicidal attempts. Among 14 primary studies from different continents, Tsegay et al. (2020) reported a lifetime prevalence of suicidal attempts of 2.19% and a 12-month prevalence of 1.64%. Lifetime prevalences of 5.5% (Kaggwa et al., 2023) and 8% (Rahman et al., 2025) were found among African and Bangladeshi medical students, respectively.

### 3.3. Risk Factors for Suicidal Ideation

Depression frequently emerged as a recurrent risk factor for suicidal ideation (R. Coentre & Góis, 2018; Kaggwa et al., 2023; Rahman et al., 2025), with two meta-analyses showing that it is the most significant risk factor among others (Kaggwa et al., 2023; Seo et al., 2021). Several studies also highlighted anxiety, burnout, poor mental health in general, and previous diagnosis of psychiatric disorders as other significant associated variables (R. Coentre & Góis, 2018; Kaggwa et al., 2023; Seo et al., 2021). In addition to psychological factors, sociodemographic and environmental variables were also found to be influential. Experiences of parental neglect, low socioeconomic status or financial strain, and substance use or addiction were commonly linked to suicidal ideation (R. Coentre & Góis, 2018; Kaggwa et al., 2023; Rahman et al., 2025; Seo et al., 2021). Female gender was notably shown to be significantly associated with increased suicidal ideation across two meta-analyses which reported on risk factors (Kaggwa et al., 2023; Seo et al., 2021), was identified as a frequent factor in one systematic review (Mateen et al., 2024), and reported to be non-significantly associated in one meta-analysis (Rahman et al., 2025). Moreover, another systematic review reported that there was some trend in the findings suggesting that females are at higher risk (R. Coentre & Góis, 2018). Academic stress, poor social support, experiences of abuse or assault, and thoughts of dropping out of medical school were also identified as common associated factors (Mateen et al., 2024; Seo et al., 2021). Clinical students were shown to have a higher risk of suicidal ideation compared to pre-clinical students in one meta-analysis (Seo et al., 2021).

### 3.4. Risk Factors for Suicidal Attempts

Only three articles reported risk factors for suicidal attempts, with two showing that depression was the only significant associated factor (Kaggwa et al., 2023; Seo et al., 2021). Both reported a high odds ratio and negligible heterogeneity for depression, indicating strong and consistent associations across multiple primary studies. The third article narratively identified factors including depression, excessive academic pressure, parental divorce and failure in the professional examination as being associated with suicidal attempts.

### 3.5. Non-Significant Associated Factors for Suicidal Ideation and Attempts

Although some risk factor results for suicidal ideation and attempts were not significant, it is worthwhile mentioning these factors for completeness. Family history of mental health disorders was shown to not be a significant risk factor for suicidal ideation in two of the meta-analyses (Kaggwa et al., 2023; Seo et al., 2021). Other non-significant factors for suicidal ideation were smoking cigarettes, parents’ high expectations, family history of suicidal behavior, and failure to pass any subject during the academic year (Rahman et al., 2025; Seo et al., 2021).

For suicidal attempts, female gender and alcohol use were both not significant risk factors in two meta-analyses (Kaggwa et al., 2023; Seo et al., 2021). Additionally, khat use and stress were also not significant (Kaggwa et al., 2023; Seo et al., 2021).

### 3.6. Recommendations for Future Research and Interventions

All the included articles discuss a need for preventative or intervention programs and strategies for medical students to help reduce the prevalence of suicidal behaviors (R. Coentre & Góis, 2018; R. M. Coentre & Figueira, 2015; Kaggwa et al., 2023; Kaur et al., 2024; Mateen et al., 2024; Peng et al., 2023; Rahman et al., 2025; Rotenstein et al., 2016; Seo et al., 2021; Tsegay et al., 2020; Wang et al., 2023; Zeng et al., 2019). Some articles emphasize a need for timely screening methods to identify struggling students as early as possible (Peng et al., 2023; Rahman et al., 2025; Tsegay et al., 2020; Zeng et al., 2019). Early detection would allow for more timely support and reduce the risk of escalation into suicidal attempts. One article recommended that future articles should use standardized interviews to evaluate variables to limit the potential under-reporting associated with questionnaires (R. M. Coentre & Figueira, 2015).

## 4. Discussion

This scoping review explored the prevalence and associated risk factors of suicidal ideation and attempts among medical students across the 12 included studies encompassing systematic reviews and meta-analyses. The findings highlight that suicidal behavior is a critical mental health issue in this population, with wide-ranging prevalence estimates that varied across different regions and timeframes. Depression consistently emerged as the most significant and frequently cited risk factor for suicidal ideation, alongside anxiety, burnout, and other indicators of psychological distress. Sociodemographic variables like gender, substance abuse, and financial difficulties were also quite significant. For suicidal attempts, only depression was significant. These results indicate that suicidal behavior is a significant mental health crisis among medical students and action should be taken as early as possible to prevent unnecessary deaths by suicide. By integrating findings across multiple reviews, this scoping review provides a global perspective on suicidal behaviors among medical students, highlights key risk factors and underexplored areas, and offers practical recommendations for interventions and policy. These insights inform the subsequent discussion on contextual factors, such as the impact of the COVID-19 pandemic, gender differences, and implications for mental health programs and interventions.

### 4.1. Before vs. After COVID-19 Pandemic Era

One meta-analysis specifically looked at the prevalence of suicidal ideation among medical students during the COVID-19 pandemic (Peng et al., 2023). The authors found that prevalences were higher during the pandemic at 15% than the pre-pandemic global prevalence of 11.1% reported in (Rotenstein et al., 2016). Notably, this trend was also observed for rates of depression, anxiety, and psychological distress (Peng et al., 2023), highlighting the pandemic’s broader impact on student mental health. Similar increases in the prevalence of suicidal ideation during the pandemic have also been noted in general populations globally (Du et al., 2023; Farooq et al., 2021), although the compounded stressors unique to medical students may have intensified these effects. Despite an increase in suicidal ideation and attempts in the general population, research on the trend in suicide rates suggest that the number of deaths by suicide remained relatively constant during the pandemic (Pirkis et al., 2022; Yan et al., 2023). Although there are no reviews directly comparing suicide rates before and after the pandemic among medical students, one could reasonably assume that the rates mirrored that of the general populations and did not significantly increase. Despite this, the apparent deterioration of the mental health of medical students as a result of the pandemic remains a significant concern and is especially important to address because these students are the future doctors who will aid others struggling with mental health issues. Any loss of life by suicide is a tragedy for all, and the loss of one’s will to live that can accompany suicidal ideation is similarly tragic. Targeting the beginnings of suicidal ideation among medical students using impactful interventions would likely stem the loss of life by suicide and largely restore the mind before the body is lost.

Longitudinal research to monitor recovery and resilience among the medical student population may be necessary to determine the long-term mental health consequences of the pandemic. Additionally, further research may be needed to assess the prevalence of suicidal ideation and its associated risk factors in the post-pandemic period among medical students. This would help determine whether elevated rates have persisted or subsided and could inform preparedness strategies for future large-scale public health crises.

### 4.2. Progression of Suicidal Ideation over Time

Understanding how medical students’ risk of suicidal behaviors progresses over the course of medical training is necessary for designing timely interventions. Clinical-phase students appear to be at higher risk of suicidal ideation than their pre-clinical peers, according to one meta-analysis which reported an odds ratio of approximately 1.7 (Seo et al., 2021). The increase in academic pressure and increased clinical exposure among other factors that accompany the transition to clinical years seems to make students more at risk of diminished mental well-being.

Among resident physicians, suicidal ideation remains a significant concern. An international survey of 1980 psychiatric residents found that around 12.3% reported active suicidal ideation, with 0.7% indicating they had attempted suicide during training (Jovanović et al., 2019). Moreover, a meta-analysis by Dutheil et al. (2019), examining suicidal ideation among physicians, found a lifetime prevalence of 17% and a 12-month prevalence of 8.6% (Dutheil et al., 2019). When compared to the pooled 11.1% suicidal ideation prevalence in medical students reported by Rotenstein et al. (2016) and 15% during the COVID-19 pandemic (Peng et al., 2023), it appears that suicidal ideation remains prevalent and may even increase into professional life. These findings highlight the fact that time alone cannot reliably increase students’ mental well-being. Without proper solutions that begin early on, the crisis will simply persist. The students then will transition to a work environment which may be more challenging, where risk factors for suicidal ideation may be even more prevalent than in the traditional medical school setting. For example, one meta-analysis reported a 28.8% prevalence of depression among resident physicians (Mata et al., 2015), which is even higher than the rate of 27.2% among medical students reported in another meta-analysis (Rotenstein et al., 2016). Higher rates of depression, since it is a prominent risk factor, could contribute to increased risk of suicidal ideation among doctors and resident physicians and potentially worsened outcomes for those who already have mental health issues. As such, there is a need for mental health interventions that begin during medical education to prevent long-term adverse outcomes resulting from a lack of timely support.

### 4.3. Prevalence of Suicidal Behavior Compared to University Students

Research indicates that suicidal ideation is a widespread concern among university students worldwide. A meta-analysis by Mortier et al. 2018 reported a 12-month prevalence of suicidal behavior among university students at 10.6%, while lifetime prevalence averaged around 22.3% (Mortier et al., 2018). Comparatively, medical students exhibit even higher rates. For instance, Rotenstein et al. (2016) found a global pooled prevalence of 11.1% over timeframes ranging from two weeks to 12 months (Rotenstein et al., 2016), while more recent estimates during the COVID-19 pandemic rose to 15% (Peng et al., 2023). This comparison highlights that while suicidal ideation is a significant concern across all university students, medical students may be at particularly elevated risk. These elevated rates may reflect certain unique pressures, distinct academic demands, or differences in help-seeking behaviors among medical students.

### 4.4. Gender Differences in Suicidal Ideation and Attempts

Male and female medical students seem to experience suicidal ideation at different rates. All three meta-analyses that reported risk factors showed that female medical students are more likely to experience suicidal ideation than males (Kaggwa et al., 2023; Rahman et al., 2025; Seo et al., 2021). Interestingly, despite an increased prevalence of suicidal ideation among females, suicide deaths are more frequent in males according to a recent systematic review which included 13 primary articles across 5 countries (Varshney et al., 2024). Around two-thirds of reported deaths were males, and around one-third were females. This higher death rate among males despite a higher ideation among females is in line with the gender paradox of suicide (Canetto & Sakinofsky, 1998). This is the idea that despite females more frequently experiencing suicidal ideation, females complete suicide at a lower rate than males do. This supports the general idea that, despite medical students being a distinct group with distinct prevalence and risk factors, the main trends affecting the general population also present themselves in this population. This is also seen from the previously stated increase in suicidal ideation during the COVID-19 pandemic.

Additionally, Tsegay et al. (2020) showed through a meta-analysis that the lifetime prevalence of suicidal attempts was higher among female medical students (7.32%) than male medical students (3.85%) (Tsegay et al., 2020). However, this study did not assess whether the difference was statistically significant. When the strength of the relationship was explicitly examined, three other meta-analyses reported no significant association between the female gender and prevalence of suicidal attempts (Kaggwa et al., 2023; Rahman et al., 2025; Seo et al., 2021). These findings indicate that while females may report higher rates of suicidal behavior in some contexts, gender alone may not be a consistent predictor of suicidal attempts among medical students. Interestingly, Tsegay et al. (2020) reported that the 12-month prevalence for suicidal attempts among medical students was 6.37% among males while it was 3.20% among females, contrary to the lifetime trend (Tsegay et al., 2020). This inconsistency across timeframes further supports that gender differences in suicidal attempts may be context-dependent and not robustly predictive when viewed in isolation.

### 4.5. Potential Risk Factors Not Explored

A broad range of risk factors were identified across many included articles encompassing important areas of risk at the institutional, societal, and personal levels, among others. Medical students are sometimes understood to be within a separate and isolated group from the general population with niche risk factors that relate to their unique experience; however, this is not exactly the case. Among the adult population, factors that are significantly associated with, or predictors of, suicidal ideation include severity of hopelessness (Ribeiro et al., 2018; Riera-Serra et al., 2024), depression (Carrasco-Barrios et al., 2020; Mudiyanselage et al., 2024; Ribeiro et al., 2018), anxiety (Bentley et al., 2016; Carrasco-Barrios et al., 2020; Ribeiro et al., 2018; Riera-Serra et al., 2024), alcohol-use disorder (Darvishi et al., 2015), sleep disturbance/problems (Harris et al., 2020; Mudiyanselage et al., 2024; Pigeon et al., 2012), childhood mistreatment/adversity (Angelakis et al., 2019; Carrasco-Barrios et al., 2020), unemployment (Carrasco-Barrios et al., 2020; Harris et al., 2020; Pigeon et al., 2012), smoking (Poorolajal & Darvishi, 2016), low social support (Carrasco-Barrios et al., 2020; Mudiyanselage et al., 2024). Most of these factors are also reported as significant risk factors for medical students, suggesting some commonality between medical students and the general adults. However, compared to the general adult population, there is much less secondary research that examines the factors associated with suicidal behavior among medical students. As a result, there is a comparatively narrower range of risk factors that are explored and summarized. Moreover, some risk factors, such as bereavement (Molina et al., 2019), multimorbidity (Xiong et al., 2020), diabetes (Elamoshy et al., 2018), and cardiovascular diseases (Chen et al., 2023), were not mentioned among factors found to be significant among medical students because they were not assessed in any of the included reviews. This presents a gap in the extent of our knowledge of risk factors specific to medical students. Without specific focus on these factors and others which may have a strong emphasis in other contexts, it is not possible to determine their impact among medical students.

Interestingly, none of the included articles assessed whether history of suicidal attempts or even suicidal ideation are significant risk factors for recent suicidal attempts in medical students, despite their significance in research of adults in multiple populations (Mudiyanselage et al., 2024; Riera-Serra et al., 2024). Although these factors seem intuitive, it is still necessary to determine the magnitude of their association as it would aid in the development of targeted and evidence-based interventions for students who are most at risk. Future research may ensure that a broad and comprehensive range of risk factors are evaluated in order to capture all possible risk factors.

For suicidal attempts, there was quite a limited number of factors assessed for significance and frequency in the included reviews. This meant that important factors may not have been highlighted, despite their emphasis in other samples. Additionally, it was noted that none of the systematic reviews or meta-analyses articles specifically focused on risk factors for suicidal attempts. These gaps can be addressed through future research.

### 4.6. Future Research Directions

All the included articles detailed the need for interventions and preventative strategies to address the increased suicidal ideation among medical students. It is important to emphasize this need, as the continued identification of the problem through more studies on prevalence is less pressing than the implementation of strategic actions that directly address the issue and help reduce the number of deaths by suicide among students. Existing research on risk factors should be used to identify students who are most at risk for developing suicidal ideation. For example, depression has frequently emerged as the most significant risk factor for suicidal ideation and attempts, indicating that students presenting symptoms may be an important focus for early identification, targeted intervention, and the development of tailored mental health support services within medical schools. The lack of intervention studies is an important gap that needs to be addressed.

More research is needed to investigate the significance of a broader range of risk factors for suicidal behaviors. Specifically for suicidal attempts, only a few factors were explored which presents a significant gap. Systematic reviews or meta-analyses could specifically focus on factors associated with suicidal attempts and evaluate a broad range of factors. For suicidal ideation, there is a much wider pool of factors, however some factors that are significant in other contexts were not explored. Future systematic reviews and meta-analysis should present a wider range of factors to ensure all possible relevant factors are assessed.

Due to the potential impact of the COVID-19 pandemic on the prevalence and correlates of mental health conditions, future studies should assess current rates and associated factors of suicidal ideation among medical students to determine whether the effects of the pandemic persist. Longitudinal research would also be valuable in examining whether these rates eventually regress toward pre-pandemic levels.

Future research should conduct systematic reviews or meta-analyses on prevalence and risk factors based on geographical locations, continents, or countries where no such review already exists. Primary studies of the prevalence of suicidal ideation exhibit drastically differing ranges across the diverse samples; hence, it would be beneficial to conduct a systematic review or meta-analysis that compiles existing data to provide a comprehensive overview that is country-specific or based on geographical locations or specific continents. This would provide a stronger form of evidence to inform region-specific policies and support the development of context-specific interventions for medical students at risk of suicidal ideation.

It is important to consider the role that under-reporting may have played in the overall prevalence reported. Research indicates that some healthcare students hesitate to seek mental health support due to fears of stigma or being perceived as weak (Al-Najdi et al., 2025; Chew-Graham et al., 2003). It stands to reason that this would increase the chances of under-reporting in self-report questionnaires. Depending on how significant this problem is across the samples, the prevalence of suicidal ideation could be significantly higher than what has been reported. Regions with greater levels of stigma towards those who struggle with mental health issues may appear to have lower levels of suicidal ideation, although this may not be the case. To reduce the rate of under-reporting, which may arise partially from the reliance on self-report questionnaires, future research would benefit from exploring other methods of data collection, such as qualitative interviews or even clinician-administered assessments.

While further research is needed to address gaps in geographic coverage, reporting accuracy, and potential effects of the pandemic, the existing evidence supports a shift from merely identifying the problem to implementing policies and support systems within medical education.

### 4.7. Limitations

This review had a few notable limitations. Only articles that had full English texts were included, which may have omitted relevant articles in other languages, limiting the interpretability of the results. Moreover, only articles published within the last 10 years were included, which may have caused relevant articles to be excluded.

### 4.8. Implications for Policy and Practice

The findings of this review underscore the need for medical schools and health education policymakers to address the high rates of suicidal ideation and attempts among medical students. With consistently high prevalence rates and well-established risk factors, there should be a focus on the implementation of mental health support systems within medical education.

Medical schools could integrate routine full mental health screening to identify students having suicidal ideations and aid in suicide prevention. Screening for suicidality would be beneficial, but it may not be the best approach to assess it as a stand-alone factor. Instead, medical schools could holistically screen for poor mental well-being among students which will help to identify not only students that are at increased risk for suicidal behaviors, but also students that generally need an increased level of support. A range of mental health disorders such as depression, anxiety, and even poor mental health in general have been reported as significant risk factors for suicidal behaviors (Kaggwa et al., 2023; Seo et al., 2021). Thus, including these factors in an assessment along with suicidal behaviors would ensure that both students having suicidal thoughts and students at higher risk for suicidal thoughts and actions can be identified so that they can be given an increased level of support. The full mental health assessment could be administered at the beginning of every semester or more frequently so that students can be checked up regularly.

Confidential counseling services and peer support initiatives are examples of specific programs that can be embedded within institutions, if not already in place, to reduce stigma and encourage help-seeking among students who are struggling. It is important to ensure that the students know that their confidentiality will be upheld, and that the information they give about their struggles will not be used against them. This would encourage more help-seeking behaviors among students for whom a lack of confidentiality would be a barrier to care.

Specific interventional programs could be designed and put into medical schools to reduce the high levels of suicidal ideation. One example of a program that was effective among medical students is one designed and implemented by researchers at the University of Hawaii John A. Burns School of Medicine (Thompson et al., 2010). The specific interventions involved increased individual, anonymous counseling for students, faculty education, and a specialized curriculum including lectures and a student handbook. The study reported on the program’s positive effect comparing pre- and post-implementation, and that the simple but comprehensive intervention was sufficient to produce significant decreases in suicidal ideation among medical students. This intervention could be replicated or modified to be used in future studies to evaluate its effectiveness across multiple larger samples.

In this technological age, confidential services could take a digital form. This would greatly increase the accessibility of these services. Supportive text message-based services like Text4Hope (B. Agyapong et al., 2023; V. I. O. Agyapong et al., 2021) have been successful at reducing suicidal ideation among subscribers compared to the control groups. A similar program could be beneficial if applied to medical students. Additionally, among medical interns, a Web-Based Cognitive Behavioral Therapy program called MoodGYM was able to produce a decreased likelihood of engaging in suicidal ideations compared to the control (Guille et al., 2015). The program involved four weekly sessions lasting 30 min each. This program or a similar one could be extended to medical students to help reduce prevalence of suicidal ideation. Past interventions that have shown effectiveness for other populations could also help guide future interventions in a medical school sample.

National and institutional guidelines can also mandate the inclusion of mental health resources as a component of medical training programs or strengthen existing resources. Moreover, institutional policies may need to be revised so that they do not discourage disclosure of mental health conditions, ensuring that students do not perceive seeking help as a professional liability.

## 5. Conclusions

In conclusion, suicidal ideation and attempts among medical students remain a significant concern globally. Addressing this issue will require more proactive and sustained commitment from medical schools and policymakers to create supportive environments that prioritize mental health and reduce stigma. Researchers can also play an important role by creating interventions and assessing their effectiveness.

## Figures and Tables

**Table 1:** Data accumulated from each study, namely the author(s) name, year of publication, continent of study population, participant characteristics, sample size, risk of bias, relevant findings, and recommendations.

Author/Date	Time Period	Number of Included Articles; Review Type	Continent of Study Population	Aim of Review	Participant Characteristics and Sample Size	Bias Assessment	Associated Factors Identified	Factors Not Significantly Associated	Key Findings on Prevalence	Other Significant Findings	Recommendations
(R. Coentre & Góis, 2018)	July 2011–May 2018.	17; Systematic review.	Europe (*n* = 3), North America (*n* = 2), Asia (*n* = 9), Africa (*n * = 2), South America (*n* = 1).	To gather recent knowledge about suicidal ideation in medical students in both Western and non-Western countries.	13,244 medical students.	None.	Depression and depressive symptoms, previous diagnosis of a psychiatric disorder, lower socioeconomic status/financial difficulties, having a history of drug use, and feeling neglected by parents were frequently associated with suicidal ideation.	-	Over the past 12 months prevalences ranged from 7% to 35.6% during medical school time from 3.7% to 4.7% current suicidal ideation ranged from 1.8% to 24.6% lifetime from 2.9% to 53.6%	There is some trend in the recent findings suggesting that females are more at risk of suicidal ideation among medical students.	Future research should look at preventive and treatment programs to reduce suicidal ideation in medical students, as well as the influence of medical school culture on suicidal ideation.
(R. M. Coentre & Figueira, 2015)	January 2005–June 2011.	10 of a total 37 addressed suicidal behavior; systematic review.	Europe (*n* = 3), North America (*n* = 5), Asia (*n* = 1), South America (*n* = 1).	To systematically review articles related to depression and suicidal behavior among medical students.	31,382 medical students among the ten studies.	None.	Not a primary focus.	-	Suicidal ideation in medical students ranged from 4.4% to 23.1%, with timeframes varying greatly between studies.	No significant relationship was found between suicidal behavior and age, or suicidal ideation and race.	Future studies should evaluate variables with standardized interviews to reduce the likelihood of under-reporting associated with questionnaires. Preventative programs such as lectures promoting general well-being should be available for medical students.
(Kaggwa et al., 2023)	Up until January 2022.	14; systematic review and meta-analysis.	Africa (*n* = 14).	To determine the prevalence of suicidal behaviors (suicidal ideation, plan, attempt, and/or suicide) among medical students in Africa.	8585 medical students.	There was publication bias based on visual inspection of the funnel plot for all three prevalences.	The factor of poor mental health, including depression, was associated with suicidal ideation and attempts in most studies. Use of addictive substances and anxiety were associated with only suicidal ideation in many of the studies The meta-analysis showed that the female gender (OR = 1.51, I2 = 19.72%), use of alcohol (OR = 2.70, I2 = 29.24%), depression (OR = 6.93, I2 =< 0.01%), and use of khat (OR = 2.40, I2 =< 0.01%) were significantly associated with having suicidal ideation and that depression (OR = 7.16, I2 =< 0.01%) was associated with suicide attempts.	The meta-analysis showed that family history of mental health disorders was not associated with suicidal ideation. The female gender, alcohol use and khat use were not associated with suicidal attempts.	The pooled prevalence of lifetime suicidal behaviors was 18.7% (I2 = 94.37%) for suicidal ideation, 3.8% (I2 = 0.03%) for suicidal plans, and 5.5% (I2 = 89.47%) for suicide attempts.	The female gender was not significantly associated with suicidal attempts in the meta-analysis.	Future research should use qualitative methods to understand the root causes of suicidal behaviors among medical students. This would allow for preventative techniques to be designed.
(Kaur et al., 2024)	January 2000–June 2023.	5 of a total of 43 studies addressed suicidal behavior; Systematic review and meta-analysis.	Asia (*n* = 5).	To provide a recent comprehensive insight into the prevalence of depression, stress, anxiety, and suicidal ideation among Indian undergraduate medical students.	1611 medical students.	No evidence of publication bias, based on the funnel plots and Egger’s regression analyses	Not a primary focus.	-	The pooled prevalence of suicidal ideation was 21% (I2 = 98%). The prevalence varied from 9% to 54%.	Analysis from the data of two studies revealed that more female students (Pooled prevalence: 34%) were affected by suicidal ideation than male students (Pooled prevalence 26%).	Institutes should implement long-term policy programs to target the root of students’ mental health issues. Additionally, counseling services should be available for all students in medical colleges.
(Mateen et al., 2024)	2015–2023.	8 of a total of 11 studies addressed suicidal behaviors; systematic reviews.	Asia (*n* = 7), Africa (*n* = 1).	To explore the causes of suicide and to mitigate psychiatric morbidity among medical students.	2454 medical students.	None.	Stressors of suicidal ideation included academic pressure, personal relationship challenges, and professional expectations.	-	Not explicitly stated.	Evidence from four studies suggests that female suicidal ideation is more prevalent than that of males. Medical students experience higher rates of suicide and suicidal ideation compared to the general population.	Development and evaluation of interventions to reduce educational stress and increase psychological well-being in medical schools should be a focus of future research.
(Peng et al., 2023)	1 January 2020–1 April 2022.	13 of a total of 201 studies addressed suicidal behaviors; systematic review and meta-analysis.	Not explicitly stated.	To evaluate the global prevalence and risk factors of several mental problems among medical students during the COVID-19 pandemic.	26,708 medical students.	Egger’s test indicated that there was no publication bias in suicidal ideation.	Risk factors described are not specific to suicidal ideation.	-	The pooled prevalence of suicidal ideation was 15% (I2 = 98.7%).	The prevalence of suicidal ideation among medical students was found to be higher than before the COVID-19 pandemic.	There is a need for timely mental screening and intervention for medical students. The revealed risk factors should be used to identify high-risk subgroups of medical students and provide targeted interventions.
(Rahman et al., 2025)	1 January 2000–May 2024.	6; systematic review and meta-analysis.	Asia (*n* = 6)	To determine the prevalence of suicidal behaviors and associated factors among medical students of Bangladesh.	1625 medical students.	Egger’s test indicated significant publication bias.	In half of the studies, the female gender, depression, familial suicidal history, and drug addiction were associated with suicidal ideation. The factors of depression and drug addiction were significantly associated with suicidal attempts. Other factors found to be related to suicidal attempts among medical students were excessive academic pressure, parental divorce, blackmailing, extramarital issues, substance use, and failure in the professional examination.	Parents’ high expectations and failure to pass any subject were found to not be significantly associated with suicidal ideation. The meta-analysis of the data from two studies showed that drug addiction/substance use (OR = 0.52, I2 = 75%) is associated with suicide and that female gender (OR = 1.24, I2 = 48%) is associated with suicidal ideation. These associations are not statistically significant.	The pooled prevalence of lifetime suicidal behaviors was 25% (I2 = 91%) for suicidal ideation, 6% (I2 = 91%) for suicidal plan and 8% (I2 = 96%) for suicidal attempt.	-	Future research should focus on employing qualitative methods to better understand the underlying causes of suicidal behaviors among medical students so that strategic preventative measures to reduce the number of deaths by suicide can be developed. Universities should employ routine screening for suicidal behaviors to allow vulnerable students to receive care.
(Rotenstein et al., 2016)	Up until 17 September 2016.	24 of a total 195 study addressed suicidal behaviors; systematic review and meta-analysis.	North America (*n* = 5), South America (*n* = 2), Asia (*n* = 9), Europe (*n* = 4), Africa (*n* = 3), Not specified (*n* = 1).	To estimate the prevalence of depression, depressive symptoms, and suicidal ideation among medical students.	21,002 medical students.	Minimal asymmetry of the funnel plot of studies involving suicidal ideation suggested a lack of significant publication bias.	Not a primary focus.	-	Crude summary prevalence of suicidal ideation reported as having occurred within the past 2 weeks to 12 months was 11.1% (I2 = 95.8%). Prevalence range was 4.9% to 35.6% between individual studies. Stratified meta-analyses showed that suicidal ideation prevalence estimates varied widely by screening tool and time frame, ranging from 7.4% (I2 = 0%) (PHQ-9, past 2 weeks) to 24.2% (I2 = 94.6%) (GHQ-28, past 12 months). Median prevalence over past 12 months reported by 7 studies was 10.2% (I2 = 96.6%).	-	There is a need for future research to investigate the root causes of emotional distress among medical students. Moreover, future studies should focus on identifying strategies to prevent and treat both depression and suicidal ideation with this population.
(Seo et al., 2021)	Up until 19 March 2021.	25; meta-analysis.	Africa (*n* = 4) Asia (*n* = 13) North America (*n* = 7) South America (*n* = 1).	To provide an overview of risk factors for both suicidal ideation and suicide attempt among medical students associated with each risk factor.	26,111 medical students.	No evidence of significant publication bias.	The meta-analysis showed that female gender (OR = 1.47, I2 = 78%), experience of parental neglect (OR = 2.53, I2 = 71%), living alone (OR = 2.15, I2= 0%), alcohol use (OR = 1.42, I2 = 54%), personal history of physical or sexual abuse (OR = 2.57, I2 = 0%), history of physical assault (OR = 3.30, I2 = 0%), comorbid mental illness (OR = 5.08, I2 = 61%), having poor social support (OR = 3.15, I2 = 47%), experiencing academic difficulty (OR = 2.23, I2 = 30%), thinking of dropping out of school (OR = 3.01, I2 = 0%), and being from a low-income status family/being in substantial debt (OR = 1.48, I2 = 55%) were significant risk factors for suicidal ideation. Additionally, it showed that clinical students were more likely to engage in suicidal ideation than preclinical students (OR = 1.71, I2 = 50%). Depression (OR = 6.87, I2 = 69%), anxiety (OR = 3.02, I2 = 0%), burnout (OR = 6.29, I2 = 69%), sleep difficulty (OR = 3.72, I2 = 75%), and stress (OR = 3.72, I2 = 92%) were also shown to be significant risk factors. For suicidal attempts, only depression was a significant risk factor (OR = 10.34, I2 = 0%).	Smoking cigarettes, family history of mental illness, and family history of suicidal behavior were not significant risk factors for suicidal ideation. Stress, female gender, and alcohol use were not significant risk factors for suicidal attempts.	Not a primary focus.	Female gender as a risk factor for suicidal attempts only trended towards significance based on the data of 3 studies.	Future research should conduct direct statistical analysis using validated scales to discern unique risk factors related to medical students versus the general population. Future research on suicidal behavior among medical students should clearly distinguish between suicidal ideation and suicide attempts, while also exploring additional potential predictors of SA to help fill existing gaps in the literature. More research on risk factors is needed so that suicide prevention programs may be constructed for medical students.
(Tsegay et al., 2020)	Up until 2020.	14; Systematic Review and Meta-Analysis.	North America (*n* = 2), South America (*n* = 1), Europe (*n* = 5), Asia (*n* = 5), Africa (*n* = 2).	To analyze the global lifetime and one-year prevalence estimates of suicidal attempts among medical students.	26,393 medical students.	no evidence of significant publication bias for prevalence of lifetime suicidal attempt. Not assessed for 12-month attempts.	Not a primary focus.	-	The pooled prevalence of lifetime suicidal attempts among medical students was 2.19% (I2 = 97.91%). The pooled 12-month prevalence of suicidal attempts was 1.64% (I2 = 87.31%).	The pooled prevalence for lifetime suicidal attempt was higher in low and middle-income countries (4.02% (I2 = 88.42%)) than for high-income countries (1.60% (I2 = 56.12%)). It was also higher for female students (7.32% (I2 = 96.20%)) than for male students (3.85% (I2 = 73.64%)). The 12-month prevalence of attempts was higher among male students (6.37% (I2 = 23.3%)) than female students (3.20% (I2 = 0.00%)). Lifetime prevalences of attempts are higher than the general population in China, Malaysia, and the general populations of Europe.	Early screening and interventions for suicidal attempts among medical students are likely needed to prevent adverse outcomes.
(Wang et al., 2023)	January 2000–December 2020.	88 of a total of 197 studies addressed suicidal behaviors; Systematic Review and Meta-Analysis	Asia (*n* = 88)	To estimate the prevalence of common mental disorders of depression, anxiety, and suicidal behaviors among medical students in China.	215,880 medical students.	There was evidence of publication bias for both prevalence of suicidal ideation and attempts.	Not a primary focus.	-	Pooled prevalence was 13% (I2 = 99.19%) for suicidal ideation, 3% (I2 = 99.01%) for suicidal attempt and 4% (I2 = 97.12%) for suicide plan.	Prevalence of suicidal ideation and attempts were highest in central China.	Formulating of effective preventative efforts and increasing the accessibility of mental health services for medical students should be the focus of future efforts. Interventional studies are also needed.
(Zeng et al., 2019)	Up until 2018.	3 of a total of 10 studies addressed suicidal behaviors; meta-analysis.	Asia (*n* = 3)	To provide a comprehensive insight into the prevalent mental health experienced by Chinese medical students as well as a basis for intervening in these problems.	3086 medical students.	The funnel plots indicated a possible publication bias.	Not a primary focus.	-	Prevalence of suicidal ideation was 11% (I2 = 97%) in Chinese medical students.	Female medical students (34%) had a relatively higher prevalence of suicidal ideation compared to male students (20%) although the difference was not statistically significant.	Longitudinal investigations with dynamic study designs are recommended for future research. Future studies are needed to investigate a wider variety of mental health problems. Timely screening and proper intervention are needed to address the prevalent mental health crisis among medical students in China.

## Data Availability

No new data was generated.
